# Allometric equations for selected *Acacia* species (*Vachellia* and *Senegalia* genera) of Ethiopia

**DOI:** 10.1186/s13021-021-00196-1

**Published:** 2021-11-02

**Authors:** Abreham Berta Aneseyee, Teshome Soromessa, Eyasu Elias, Gudina Legese Feyisa

**Affiliations:** 1 College of Agriculture and Natural Resource, Department of Natural Resource Management, Wolkite University, P. O. Box 07, Wolkite, Ethiopia; 2 Center for Environmental Science, College of Computational and Natural Science, Addis Ababa University, P. O. Box No: 1176, Addis Ababa, Ethiopia

**Keywords:** Above ground biomass, *Acacia* species, Allometric biomass equation, Carbon stock, Omo Gibe woodland

## Abstract

**Background:**

Allometric equations are used to estimate biomass and carbon stock of forests. In Ethiopia, despite the presence of large floral diversity, only a few site-specific allometric equations have been developed so far. This study was conducted in the Omo-Gibe woodland of south-western Ethiopia to develop an allometric equation to estimate the Above-ground Biomass (AGB) of the four *Acacia* species (*Senegalia polyacantha*, * Vachellia seyal, Vachellia etbaica* and *Vachellia tortilis*)*.* Fifty-four (54) *Acacia* trees were sampled and measured within 35 temporarily established square plots. In each plot, dendrometric variables were measured to derive the models based on combinations of Diameter at Breast Height (DBH), height, and wood density as predictor variables. Model performance was evaluated using goodness-of-fit statistics. The biomass was compared using four allometric biomass models that have been widely used in the tropics.

**Results:**

The model containing DBH alone was more accurate to estimate AGB compared to the use of multiple predictor variables. This study, therefore, substantiated the importance of site-specific allometric equations in estimating the AGB of *Acacia* woodlands. This is because a site-specific allometric equation recognizes the environmental factors, vegetation types and management practices.

**Conclusions:**

The results of this study contribute to a better understanding of allometric equations and an accurate estimate of AGB of *Acacia* woodlands in Ethiopia and similar ecosystems elsewhere.

**Supplementary Information:**

The online version contains supplementary material available at 10.1186/s13021-021-00196-1.

## Background

Allometric equations are widely used to estimate forest carbon stocks (C) [1]. Accurate estimates of biomass are crucial for the assessment of carbon stocks toward understanding carbon variations in response to world climate changes [2, 3], monitoring ecological processes such as wood production and nutrient cycling [4] and sustainable forest management [5].

Currently, various mechanisms have been proposed by the United Nations Framework Convention on Climate Change (UNFCCC) to reducing carbon emissions for climate change adaptation and mitigation. The major climate mitigation option is Reducing Emissions from Deforestation and Degradation (REDD^+^) by promoting conservation, sustainable management of forests, and enhancing forest carbon stocks in developing countries [6]. One of the critical elements for the REDD^+^ mechanism is the capacity to know the carbon storage potential of forest ecosystems [7]. This can be achieved by establishing biomass allometric equations to reduce uncertainties in carbon accounting and carbon trading in the voluntary and mandatory markets [8, 9].

Different methods, ranging from the most generic to the site and species-specific allometric equations are available for estimating the biomass of a tree [10]. Although the general allometric equations are used widely, they may not be able to predict local biomass accurately due to differences in the tree architecture such as several stems, height, age, diameter, stand density, cultivar, site conditions (climate and soils), and management practices [11, 12]. For example, pruning and coppicing can affect the rate of biomass accumulation [13] as well as the change in biomass without changing the Diameter at Breast Height (DBH) [14, 15]. The model has shown a large prediction error based on the findings of Mugasha et al. [16] and Kachamba et al. [17] in miombo woodlands, Tesfaye et al. [18] and Ubuy et al. [19] in dry forests, and Lisboa et al. [20] in moist forests. Therefore, such types of generic equations may show a systematic error of up to 400% at the site level [15].

Site-specific models provide less bias than general models [21] because the local climatic, soil properties, altitude, and land-use history are affected by tree growth characteristics [22]. According to Mokria et al. [23], the lack of a site-specific allometric model for estimation of Above Ground Biomass (AGB) is the key reason for persistent inaccuracy and low uncertainty in biomass estimation in sub-Saharan Africa. Solomon et al. [24] also stated that the precise estimation of biomass and carbon stock in a forest can be achieved using site-specific allometric equations for the species and forest types.

In their review of biomass models in sub-Saharan Africa, Henry et al. found 63 models from Ethiopia [23]. These models included only six species and 70% were for Eucalyptus ssp. More recently, many authors have developed local species-specific allometric models to estimate tree AGB for different parts of Ethiopia [16, 23, 25–28]. However, they are not representative of all vegetation types and agro-ecosystems of Ethiopia.

Most of the previously developed allometric equations were based on the destructive method, which is costly and time-consuming to implement in the wide strata of a forest area [29, 30]. According to Henry et al. [31], the allometric equations in Ethiopia are developed based on a destructive sampling method, which does not obey environmental principles and is not feasible in large-scale analysis [29]. There are also cultural, legal and policy aspects of applying the destructive method for tree sampling. In Ethiopia, the national legislation does not allow logging activities of the indigenous trees. According to Tesfaye et al. [18] cutting down an indigenous tree is prohibited by law, and this made it difficult to develop biomass allometric equations for the species. Recently, a semi-destructive method has been explored using Picard et al. [32] and satellite data [33] procedure. The semi-destructive method can also help for easy measurement of the parameters without cutting the tree. Therefore, the semi-destructive sampling method was used for modeling the AGB of the indigenous trees, since it can be applied to degraded woodlands and in key conservation areas where cutting is prohibited [33]. Publications on semi-destructive methods have been increasing in recent years in Ethiopia such as Wof-Washa dry Afromontane forest in Ethiopia [25], Biosphere Reserve forest of southwestern Ethiopia [27] and Mana Angetu moist Afromontane forest of Ethiopia [28] and Hill zone of Bangladesh [34].

In Ethiopia, several forest resource assessment campaigns have been undertaken to understand the AGB status and management options of the forest resources [35]. However, achieving high-level accuracy of these assessments estimation remains to be challenging [23]. This is partly because of a high level of uncertainty emanating from the nature of the allometric models used for biomass determination. Measurement errors and sample sizes are also major sources of uncertainty in the AGB assessment efforts [36, 37]. It is clear that site and species-specific models need to be developed and the suitability of the existing allometric equations needs to be evaluated to make accurate biomass assessment and make an informed decision. Several allometric equations are claimed to be suitable for ‘tropical forests. However, tropical forests are highly diverse in their ecology and vegetation composition, ranging from moist high forests to woodlands in arid climates. The high biological diversity of tropical forests also makes it difficult to use a ‘one-fits-all’ model. Particularly the *Acacia* woodlands, which are ecologically and economically of high value in Ethiopia, are given little research attention from the point of view of developing allometric models that are suitable for accurate forest biomass assessments.

*Acacia* woodland is an iconic ecosystem in Ethiopia, accounting for 11% dry land woodland of the country [38]. In this case, a woodland is defined as the type of land cover characterized by trees and shrubs with a tree crown cover of 5–10% of trees able to reach a height of 5 m at maturity and a crown cover of more than 10% of trees not able to reach a height of 5 m at maturity, having fewer species diversify [39]. More than 58 *Acacia* species are known to occur in Ethiopia, of which 49 are indigenous [40] and six of these are threatened species [41]. *Acacia (Vachellia and Senegalia genera)* is widely distributed in the Omo Gibe valley of Ethiopia [42] and it provides paramount ecosystem services values, including food and habitats for a variety of animals, from hoofed mammals and birds to countless species of insects [43], nitrogen-fixing, and hydrological regulation.

Therefore, this research was set out to develop a species-specific biomass allometric equation for estimating the AGB of the *Acacia* species (*Vachellia* and *Senegalia* genera) in the woodland of Omo Gibe valley in Ethiopia. In fact, it is genus-specific because it included different Acacia species.

Thus, this study contributes to the reliable estimate of AGB in the woodland ecosystems and contributes to informing decisions on woodland management and carbon accounting, further facilitating carbon trading and global climate change mitigation. The objectives of the study were, therefore, to: (1) derive and evaluate AGB models for selected *Acacia* species (*Vachellia* and *Senegalia* genera) in Omo Gibe valley, and (2) to compare performances of the widely used biomass equations in estimating biomass of *Acacia* woodland.

## Methods

### The study area

**Fig. 1:**
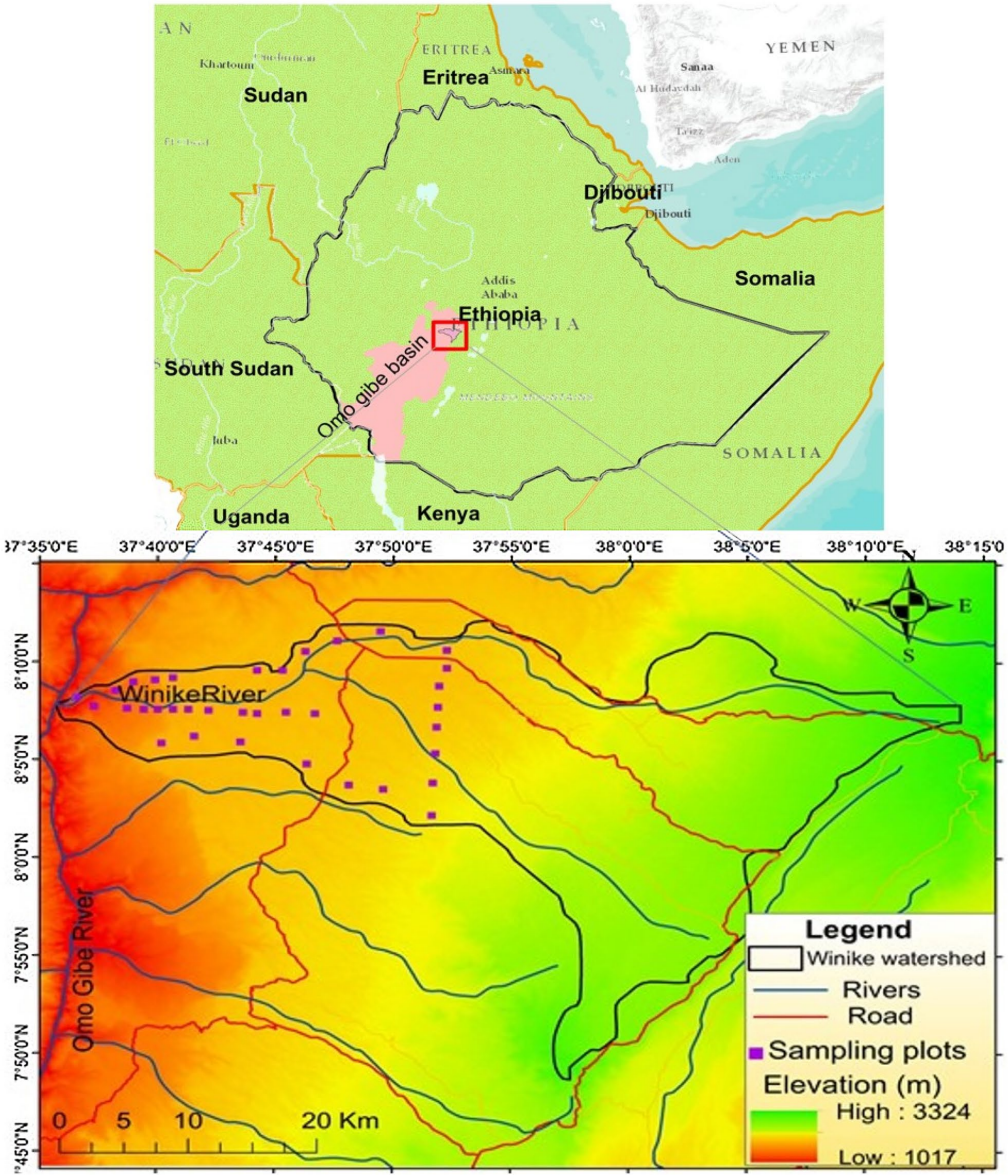
The geographic location of Omo Gibe woodland in Ethiopia. The map was produced in ArcGIS Desktop version 10.4 (https://www.esri.com/en-us/arcgis/products/arcgis-desktop/)

The Omo-Gibe woodland is located in the Omo gibe Basin, part of central highlands Ethiopia and 210 km away from Addis Ababa, the capital city of Ethiopia. The geographical bound of the area is within the coordinates of 037° 40′–038° 10′ longitude E and 07° 50′–08° 20′ N latitude (Fig. 1). It is situated in the headwaters of the Om-Gibe basin which is one of the most important river basins in the country. The elevation ranges between 1096 m and 2153 m, and the mean altitude is 1735 m. The area is characterized by a wide range of topographic features, ranging from deep gorges (faults), through dissected ridges to undulating plateaus. Due to the altitudinal differences, there is a large variation in rainfall and temperature year to year in the study area [44]. The rainfall increases with altitude but temperature decreases [45]. Based on Ethiopian Meteorological Agency (EMA) data analysis, the annual average rainfall varies from 856 to 1600 mm, with a bimodal distribution that allows two growing seasons [45, 46]. The main rainy season (monsoon rains) extend from June to September and the short season (*Belg* rains-spring) fall between March and April. The mean minimum and maximum temperatures are 12.7 °C and 26.7 °C, respectively.

The *Acacia* woodland of the Omo Gibe valley covers about 3,179,060 ha (40.02%) of the total land area [47]. The dominant species are *Senegalia senegal* (L.) Britton.*, Vachellia seyal* (Delile) P.J.H. Hurter*, Vachellia tortilis* (Forssk.) Galasso & Banfi*, Senegalia polyacantha* (Willd.) Seigler & Ebinger*, Vachellia etbaica* (Schweinf.) Kyal. & Boatwr., *Acacia mellifera* (Vahl) Benth*, Commiphora africana* (A. Rich.) Engl., *Commiphora myrrha* (Nees) Engl., *Cordia monoica* Roxb., and *Commiphora boranensis* (Voll). The area is among the most densely populated areas of the country, (283 people per square kilometre) and the major economic source is agricultural activities [42].

### Biomass data collection method

A forest inventory was carried on 35 temporarily established plots (50 × 50 m) using a systematic random sampling procedure. *Acacia* species were identified in the field using the tree identification manual developed by Bekele et al. [48] and with the assistance of a skilled botanist. The samples were established based on the Nyman optimal allocation formula [49]. All trees with DBH > 10 cm within each plot were identified and DBH measurement of these trees was undertaken. Trees were stratified into 6 DBH classes for biomass analysis (Table 1): < 20, 20.1–30, 30.1–40, 40.1–50, 50.1–62.1, and > 62.1 cm. For the AGB model development, from each DBH class, two individual tree samples were taken from the three *Vachellia* genera (*Vachellia seyal, Vachellia tortilis,  Vachellia etbaica)* (6 DBH classes × 2 trees × 3 genera  = 36 samples), and three tree samples were taken from one genera of *Senegalia (Senegalia polyacantha* (6 DBH classes × 3 trees × 1 genera = 18 samples)). In total, 54 tree samples were selected and tagged properly. In case a plot was more diverse than others, we took two samples. In each sampling plot, DBH (cm) above ground from the uphill side of the standing tree was measured using a caliper and the total height of the tree was measured by climbing up at the top of the tree using a measuring tape. Moreover, the diameter and height of the untrimmed branches were measured in the field. Also, fresh biomass of trimmed small branches and leaves were measured in the field before sending the samples to the laboratory. The trimmed small branches and untrimmed branches were differentiated based on their diameter: trimmed small branches are those that have diameter < 10 cm and these were harvested (trimmed) for the analysis in the laboratory whereas untrimmed branch diameter > 10 cm. Moreover, we counted the number of large and small branches in the standing marked tree to calculate biomass for each of tree components later.

**Table 1:** DBH class distribution (tree/ha) of *Acacia* species

Species	DBH classes (cm)						Total
	< 20	20.1–30	30.1–40	40.1–50	50.1–62.1	> 62.1	
*Vachellia seyal*	36	45	34	37	25	14	191
* Vachellia tortilis*	21	40	29	31	18	17	156
*Vachellia etbaica*	18	32	42	30	37	19	178
*Senegalia polyacantha*	20	27	35	47	72	60	261

### Biomass calculations

The total AGB of the standing tree was partitioned into four architectural components to apply the semi-destructive approach. These are dry sections (stem and untrimmed large branch), untrimmed small branch, trimmed small branch (SB), and leaves (Table 2). According to Picard et al. [32], the procedure for obtaining the biomass of stem and the untrimmed large branch is the same. The dry weight of the large branches and stem (dry section) was obtained by using Eq. (1).

1 $$B_{dry section} = {\overline{\rho }} \times {\text{Vi}}$$

**Table 2:** Total biomass and tree components dendrometry variables of *Acacia* species (n = 54)

Variables	*Senegalia polyanatha*			*Vachellia seyal*			*Vachellia etbaica*				*Vachellia tortilis*	
	Mean	Max	Min	Mean	Max	Min	Mean	Max	Min	mean	Max	Min
Dry section	2796 ± 184	4648	1575	2102 ± 172	3780	1000	2084 ± 152	3480	1250	2321 ± 157	5000	482
U_SB_	7 ± 0.5	11	4	7 ± 0.7	10	3	2.6 ± 0.5	4	1	3 ± 0.5	6	1
T_SB_	1 ± 0.01	2.3	0.3	1 ± 0.01	1.19	0.07	1.1 ± 0.02	1.8	0.5	0.8 ± 0.07	1.4	0.5
Leave	0.09 ± 0.02	0.83	0.02	0.13 ± 0.02	0.23	0.07	0.15 ± 0.02	0.12	0.02	0.4 ± 0.02	0. 78	0.12
DBH	101 ± 5.6	155	14	76 ± 3.5	130	11	87 ± 4.05	91	12	77 ± 4.5	143	12
H	13 ± 1.3	22	7	8 ± 1	20	6	11 ± 1.6	19	8	10 ± 1	17	8
Volume	8 ± 0.7	16	4.5	5 ± 0.8	7	3.5	6 ± 1.2	8	3.5	6 ± 0.8	10	4
ρ (g/cm^3^)	0.36 ± 0.02	0.5	0.2	0.45 ± 0.03	0.63	0.25	0.38 ± 0.03	0.6	0.18	0.36 ± 0.04	0.58	0.12
AGB	2804	4649	1584	2087	3483	1252	2643	4649	1252	2711	4649	1252

Dry section: Stem and large untrimmed branch (diameter > 20 cm); U_SB_: Untrimmed small branch ((diameter 10–20 cm); T_SB_: Trimmed small branch (diameter < 10 cm); DBH: Diameter breast height; H: Height; volume: Volume of dry section (stem and untrimmed large branch); AGB: Total above-ground biomass and $\rho$: Wood density

where, $B_{dry section}$ = biomass of the stem and untrimmed large branch, Vi = Volume of section i and ${\overline{\rho }} =$ density of wood (obtained from the ratio of the biomass of trimmed small branch to its volume, determine in the laboratory).

The volume (m^3^) of the stem and untrimmed large branches were calculated (Eq. 2) from the cross-sectional measurements using Smalian’s formula [50], assuming that each of the measured stem and the large untrimmed branches was a cylindrical shape. The stem and the untrimmed large branches in each tree were subdivided in the section at the intervals of 2 m distance and the top end and the lower end of each section interval diameter was measured to obtain the volume of each section. Since the division was short (2 m), the shape of the sections was a bit different from a cone, with very little tapering; thus, volume determination using Smalian's and other formulas may have very slight differences [32]. Therefore, the mean volume of the stem and untrimmed large branch (dry section) was determined by the sum of the volume of each section’s diameter measurement at 2 m interval divided by the number of the section in the trunk and untrimmed large branch.

2 $$V_{i} = \frac{\pi }{8}Li\left( {D_{li}^{2} + D_{2i}^{2} } \right)$$

where, V_i_ = the volume of section i, Li is its length of the trunk and untrimmed large branch (m), and D_1i_ and D_2i_ are the diameters (cm) of the two extremities of section i. (i.e., D_1i_ = diameter of the narrow end of the trunk (cm) and D_*2i*_ = diameter of the large end of the trunk (cm)).

### Biomass of untrimmed small branch

The untrimmed branches are those that have a diameter > 10 cm and these were not cut down in a standing tree. The biomass of the untrimmed small branch was calculated using a linear regression equation between the diameter of the untrimmed small branch and biomass of the trimmed small branch (Eq. 3). The linear relationship was first established based on biomass measurements from trimmed small branches and the corresponding diameter of the untrimmed small branch. We then used the parameter estimates from the linear regression and used to estimate the untrimmed small branch biomass. To determine the total biomass of untrimmed small branches per tree, the calculated biomass of the untrimmed small branches were multiplied by the number of untrimmed small branches.

3 $${\text{B}}_{{{\text{untrimmed biomass}} {\text{small branch}}}} = {\text{ a}} + {\text{bD}}$$

where, B = the biomass of the untrimmed small branches and D = the untrimmed diameter of the small branch (measured), a, b are model parameters (intercept and slope, respectively).

### Biomass of trimmed small branch and leaves

The small branches of trees were trimmed using the local machete and the twigs and attached foliage were carefully separated from each trimmed small branch. Two small branches were trimmed for sampling from each tree for laboratory analysis. To determine the fresh weight of the trimmed small branch (wood aliquot) and leaves of each compartment, their biomass was measured using a spring hanging weighing scale of 5 kg capacity (0.02 kg precision).

The total biomass of the trimmed small branches was obtained from the number of small branches in the standing tree multiplied by the average biomass of the trimmed branch. Similarly, to obtain the total biomass of the leaves of a tree, the average leave biomass was multiplied by the total number of small branches per tree.

### Laboratory analysis

Fresh sub-samples of 200–250 g for trimmed small branches and 150 g for leaves of each marking tree were transported to the laboratory to determine the moisture content, wood density and volume of the trimmed components. For wood density (g/cm^3^) and volume (cm^3^) analysis, disc samples were cut using a hand saw from each trimmed small branch, and fresh weights of these samples were measured using spring hanging. The total number of discs were 72: two discs per species (36 for *Senegalia polyacantha* and 36 discs for the three species altogether (*Vachellia seyal, Vachellia etbaica, Vachellia tortilis*)) and the Wolkite University laboratory was used for the analysis. Wood aliquot volume was determined using the water displacement method, as outlined in Vieilledent et al. [51]. The aliquots were then oven-dried (105 °C for 72 h.) to determine the moisture content. The determination of wood density for each ith discs collected from the j^th^ sampled trees were estimated from the ratio of oven-dry weight (g) and volume (cm^3^). The mean wood density ($\rho$) for each tree was computed using Eq. 4.

4 $$\rho = \mathop \sum \limits_{j = 1}^{j = 72} \rho_{j} /n$$

where n = the number of samples, j = disc samples from 72 trees.

The total AGB (kg/tree) of a standing tree is, therefore, the sum of all components biomass of the tree. That is the sum of the biomass of the dry section, untrimmed small branch, and trimmed small branch and leaves (Table 2).

### Construction and evaluation of allometric equations

Before establishing the allometric equation, data were evaluated for outliers and then the sources of these outliers were investigated. Eventually, measurement errors were detected to be the source of the outlier data. The outliers were, therefore, removed and data were collected again to replace the wrong and removed data. Six non-linear regressions were used to test the best-fit model for the total AGB and tree components biomass such as dry section, untrimmed small branch, trimmed small branch and leaves.

The models and the corresponding variables used were: Model 1 (DBH only), Model 2 and 6 (DBH in combination with height), Model 3 (DBH in combination with density of wood), and Model 4 and 5 (DBH combination of H and ${\uprho }$) (Table 3). The statistical analysis was conducted in R software, using a package of ‘NLS tools’[52].

**Table 3:** Models used for estimating the AGB of *Acacia* species

Model	Combination of variables
1	$AGB = \alpha \times \left( {DBH} \right)^{{{\upbeta }_{1} }}$
2	$AGB = \alpha \times DBH^{{\beta_{1} }} \times H^{{\beta_{2} }}$
3	$AGB = \alpha \times DBH^{{\beta_{1} }} \times {\uprho }^{{\beta_{3} }}$
4	$AGB = \alpha \times DBH^{\beta 1} \times H^{{\beta_{2} }} \times {\uprho }^{{\beta_{3} }}$
5	$AGB = \alpha \times (DBH^{2} \times H \times {\uprho })^{\beta 3}$
6	$AGB = \alpha \times DBH^{2} \times H^{{\beta_{2} }}$

AGB: Total above-ground biomass of tree (kg/tree), $\alpha$: Intercepted, DBH: The diameter at the breast height (cm), H: Height (m) and ρ: Wood density (g/cm^3^) and $\beta_{0} \beta_{0} , \beta_{1} , \beta_{2} \, and \, \beta_{3}$ are the regression coefficient attributed to their scaling parameter

Model diagnostics were performed using goodness-of-fit statistics, namely, Corrected Akaike Information Criterion (AICC) (Eq. 5), Root Means Square Error (RMSE) (Eq. (8)), and Residual Standard Error (RSE) [53]. The best species-specific biomass allometric equation showed the lowest AICC and Residual Standard Error (RSE) [17]. Based on these diagnostics, the models were ranked (1 to 6) according to each goodness-of-fit statistic [54]. The RMPE and MPE were conducted in excel using the corresponding Eqs. 6 and 7, respectively, which were used to show the models performance. All the advanced statistical analyses such as AIC and RSE were done using R software.

Model validation was carried out using the leave-one-out cross-validation (LOOCV) procedure [55] and results were assessed using the adjusted coefficient of determination (R^2^_adj_) and Root Mean Square Error (RMSE) statistics, using Eqs. 8 & 9. The LOOCV followed the algorithm as reported by Ji et al. [56]. A single plot was withheld as a validation sample and the remaining plots were used to train the models. The advantage of the LOOCV technique is providing an unbiased estimation of the prediction error and to increase the robustness in the results [57].

5 $$AICC = nlog\left( {\mathop \sum \limits_{i = 1}^{n} \frac{{\left( {AGB_{est,i} - AGB_{obs,i} } \right)^{2} }}{n}} \right) + 2p$$

6 $$RMPE = \mathop \sum \limits_{i = 1}^{n} \frac{MPE}{Y} \times 100$$

7 $$MPE = \frac{{\left( {AGB_{est,i} - AGB_{obs,i} } \right)}}{n}$$

8 $$RMSE_{i} = \sqrt {\frac{{\mathop \sum \nolimits_{j = 1}^{n} \left( {obs,_{i} - mean est,_{i} } \right)^{2} }}{n}}$$

9 $$R^{2} Adj = \frac{{1 - \sum (obs,_{i} - mean est,_{i} )^{2} }}{{\sum (obs,_{i} - mean est,_{i} )^{2} }}$$

where, AIC (unitless) = Akaike’s Information Criterion, RMPE = Relative Mean Prediction Error (%), RMSE = Root mean Square Error, MPE = Mean Prediction Error (%), R^2^ = Adjusted coefficient of determination,$AGB$ est,*i* = Predicted AGB and $AGB$ *obs*,*i* = Observed total AGB of individually measured tree i and Y = the average of observed total AGB (kg/ tree), n = the total number of sampled trees, and p = the number of parameters in the tested model.

### Comparison of biomass allometric equation

A comparison of the allometric equation was undertaken to estimate AGB (Table 4). A large number of regression models have already been published; however, this study focused on four equations, which are widely used in Ethiopia for biomass assessment. The four equations considered in our comparisons are Brown [58] allometric equation, which was developed based on data from the moist tropical forest; Henry et al. [31], who used data from the dry land forest of East Africa; Chave et al. [15], using data from the tropical dry forest and Guedes et al. [59] from the dry land ecosystem in Mozambique. We compared the performance and validity of the allometric equations developed in Omo Gibe woodland comparing with the four published allometric equations using MPE and RMPE [17]. For validation, the AGB collected in the field (observed) and the predicted biomass using the existed allometric equations were used.

**Table 4:** Suggested published allometric models used to compare the study site-specific allometric equation

Suggested allometric equations	Sources	DBH (cm)	Location and forest types
1. AGB = exp (− 2.134 + 2.430 × ln(DBH))	Brown [58]	4–148	Moist forest (pantropical)
2. AGB = 0.0673 $(\rho$(DBH)^2^H)^0.976^	Chave et al. [15]	5–156	Dry forest (pantropical)
3. AGB = 0.0983 + 0.000002 * (DBH)^2.4307^ *(H)^1.5607^	Henry et al. [31]	1–200	East Africa dry forest
4. AGB = 0.1754 × DBH^2.3238^	Guedes et al. [59]	5–53	woodland in Mozambique

## Results

### Tree density

To understand the AGB per hectare, tree density (number of trees per unit area of land) analysis was conducted and accordingly, *Senegalia polyacantha* was found to have higher tree density (261 ± 2.3 trees/ha), followed by *Vachellia seyal* (191 ± 1.5 trees/ha) and *Vachellia etbaica* (178 ± 1.2 trees/ha). The *Vachellia tortilis* was found to be of lower tree density (156 ± 1.1 trees/ha) (Table 1).

### AGB estimates and models

The observed mean AGB in the Omo Gibe woodland was 2397 ± 192 kg/tree. The mean AGB for *Senegalia polyacantha*, *Vachellia tortilis*, *Vachellia etbaica* and *Vachellia seyal* was 2804 ± 165 kg/tree, 2326 ± 154 kg/tree, 2087 ± 133 kg/tree, and 2111 ± 142 kg/tree, respectively (Additional file 1: Table S1). Based on goodness-of-fit statistics and LOOCV analysis, the model based on DBH alone (y = αDBH^β^ in model 1) provided the best fit for AGB estimation. Across all equations, the parameter β_1_ describing the influence of DBH was statistically significant (*p* = *0.0041*) in each model. Similarly, the model parameter β_2_ describing the influence of height was also statistically significant (*p* = *0.0053*). The parameter β_3_, showing the influence of wood density, however, was not significant (*p* = *0.0072*) (Additional file 1: Table S2).

**Fig. 2:**
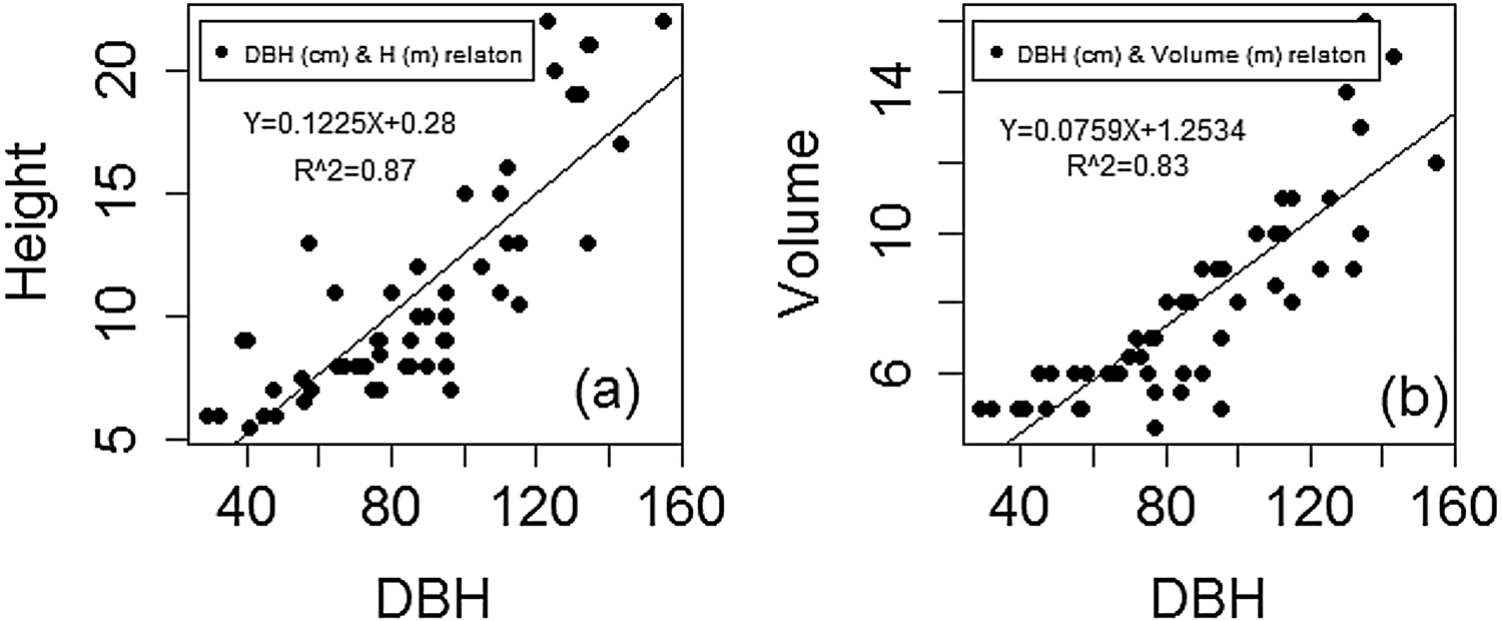
Diameter relationship with height (**a**) and volume (**b**)

The AGB of each tree component expressed as a function of DBH only in model 1 had a lower AIC (494) and RSE (52 kg/tree), which indicates a very good performance of the model. The performance of Model 2 in estimating AGB was shown to be second to that of Model 1 and the influence of DBH and height in predicting AGB (parameters β_1_ and β_2_) were significant (*p* = *0.0076*), with AIC and RSE values of 501 and 61 kg/tree, respectively, (Additional file 1: Table S2). Model 6 ranking third, having AIC and RSE values of 525 and 74 kg/tree, respectively. Model parameter β_3_ was, however, non-significant (*p* = *0.182*). Models 4 and 5 had approximate AIC and RSE values and ranked the fourth and fifth, respectively. Model 3 had the highest AIC and RSE, ranking 6^th^. The model parameter of wood density (β_2_) was non-significant (*p* = *0.063*). Adding wood density as a predictor variable in the model with a combination of DBH and height did not improve the performance of the models. Correlation analysis of predictor variables shows that there is a strong relationship between DBH and tree height (*R*^*2*^ = 0.87, *r* = *0.*83) (Fig. 2a) and volume (R^2^ = 0.83, r = 0.77) of the trees (Fig. 2b).

The LOOCV validations among the six evaluated models confirm that the similarity and highly reliable relationships between predicted and observed data for the models, with no substantial trends in the prediction errors. For each model prediction, the cross-validation revealed no differences between RMSE and RMSEcv (Table 5). The adjusted R^2^ also did not show a significant difference between the normal and the LOOCV analysis of the models.

**Table 5:** leave-one-out cross-validation (LOOCV) of AGB estimation

Model types	RMSE-norm	R^2^ adj-norm	RMSE cv	R^2^ adj cv	R^2^ adj difference	RMSE difference
model 1	0.82	0.85	0.9	0.88	0.03	0.08
model 2	1.31	0.83	1.38	0.87	0.04	0.07
model 3	1.92	0.7	2.13	0.78	0.08	0.21
model 4	1.53	0.73	1.74	0.84	0.11	0.21
model 5	1.56	0.74	1.66	0.82	0.08	0.1
model 6	1.4	0.77	1.49	0.83	0.06	0.09

RMSE norm: Root-mean-square error normal; RMSEcv: Root-mean-square error for cross-validation; R^2^_adj_-norm:  Adjusted R square normal; R^2^_adj_ cv: Adjusted R square for cross-validation

### Comparison of allometric equations

**Fig. 3:**
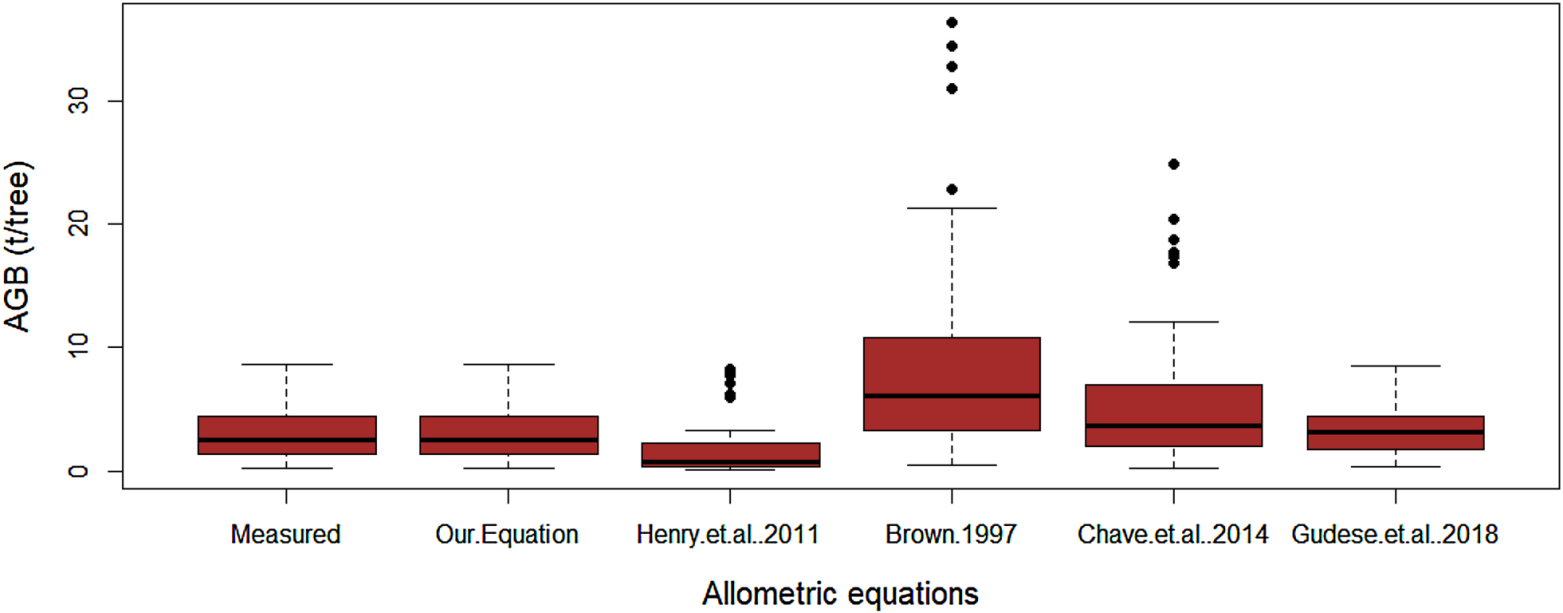
Box plot comparison of biomass in the study site and previously published models

**Fig. 4:**
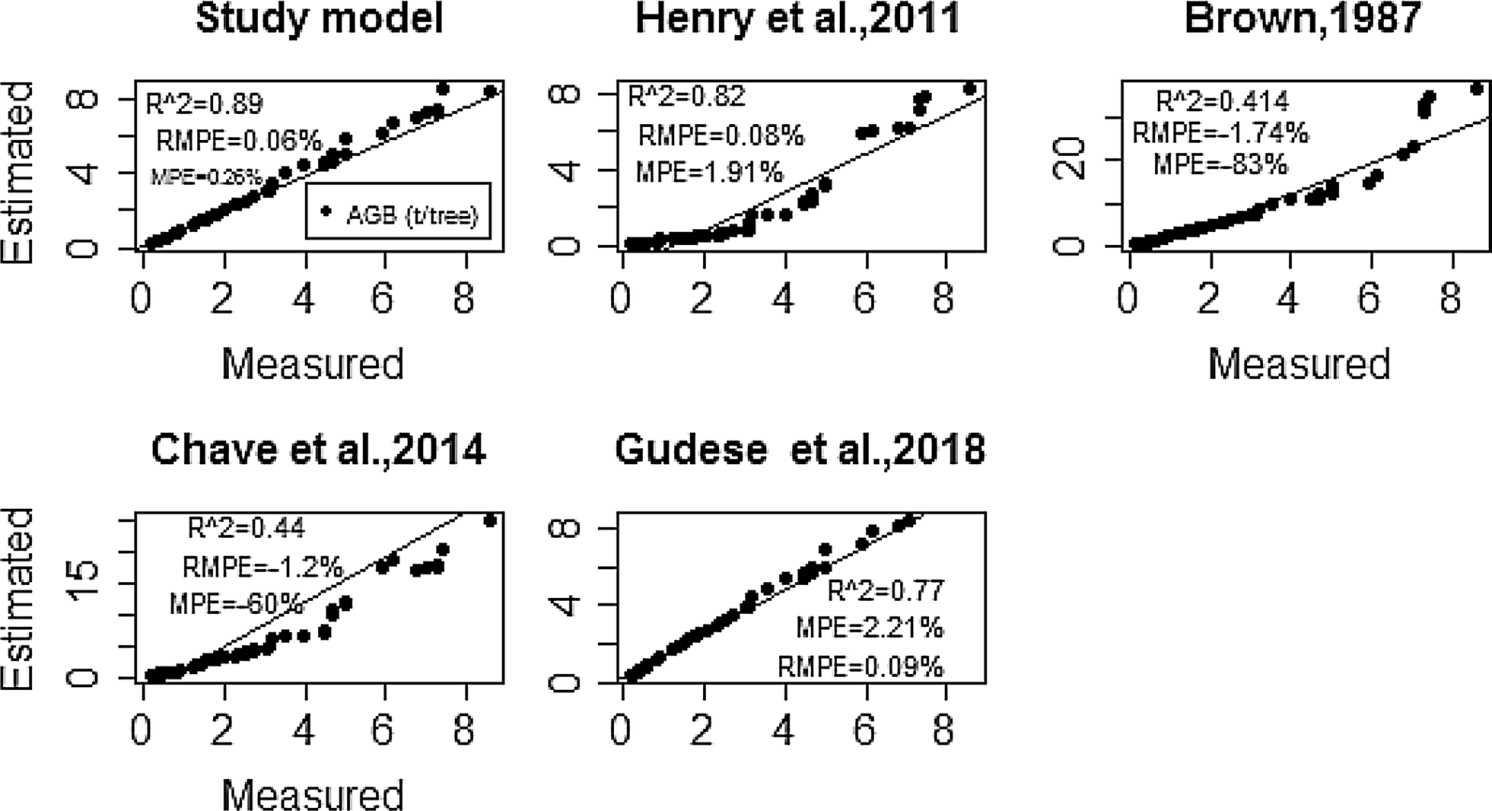
Relationships between estimated and measured AGB of sample trees in tone (t) per tree (*n* = 35). R^^2^*, RMPE% and MPE%* show error produced in the estimation of the biomass

The average AGB estimate based on the equation developed in this study was 2940 ± 195 kg/tree. Estimates based on Henry et al., and Guedes et al., models were 2521 ± 171 kg/tree and 3240 ± 203 kg/tree, respectively. The pan-tropical models developed by Chave et al. [15] and Brown [58], which are currently being widely used in Ethiopia for biomass and carbon stock assessments, are shown to have a large variability of prediction and overestimating the AGB of *Acacia* woodland in the study area (Fig. 3). Statistically, no significant difference (*p* = *0.0072*) was observed among the AGB estimate based on our model and those based on Henry et al. [31] and Guedes et al. [59]. However, a comparison of our model with that of the Pan-tropical models: Brown [58] and Chave et al. [15], shows a significant difference in AGB estimates (Fig. 4).

The comparison of the allometric equations showed a large variation of accuracy in terms of RMPE and MPE. The RMPE and MPE value of the allometric equations developed in this study was 0.06% and 0.26%, respectively. The RMPE and MPE values for Guedes et al. [59] and Henry et al. [31] were close to those calculated for the equations developed in this study (respectively, RMPE and MPE values of 0.08%, and 1.91% for Henry et al. and 0.09% and 2.21% for Guedes et al. The largest RMPE and MPE values were those calculated for Brown [58] (respectively, − 1.74% and − 83%), and followed by Chave et al. [15] (RMPE and MPE of-1.2 and − 60%, respectively). These values were by far higher than the values obtained using the allometric equation developed in this study.

## Discussion

In this study, we evaluated the performance of allometric equations to estimate tree AGB in Acacia woodlands using multiple predictor variables (DBH, Height (H) and wood density).In agreement with many similar studies, our findings demonstrated that the use of DBH alone would provide a more accurate estimate of the total AGB than using two or more parameters which is in agreement with many other studies [15, 24, 60, 61]. This is convenient because DBH is a variable that is easy to measure, compared to wood density and height. Height and wood density is, perhaps, more prone to measurement errors than DBH, which could influence model performances. Contrary to our findings, several other studies reported that the inclusion of multiple predictors, such as wood density, in combination with DBH and height, provides a better estimate of tree biomass [62–64]. However, due to the time-consuming nature and high cost involved in measuring height and wood density, the practical applicability of these variables in biomass assessment of a large tract of forest areas could be limited.

The correlations investigated show that AGB was significantly and positively correlated with DBH (r = 0.81) and height (r = 0.59) but not with wood density (r = 0.069) (Additional file 1: Table S3). The lack of correlation between the AGB and wood density indicates that wood density is not considered a potential-dependent variable in the model. Based on the global wood density database, wood density can vary widely among individual trees within a species in a given region [65]. Wood density can vary depending on tree density and environmental factors such as climate, soil type and altitude [59]. Therefore, according to Zanne et al. [65] and Chave et al. [66], the wood density ranges from 0.1 to 1.5 g/cm^3^ among tropical trees and the *Acacia* species wood density in Africa also ranged from 0.48 g/cm^3^ to 0.826 g/cm^3^, with an average of 0.612 g/cm ^3^ [67] whereas, in this study, the *Acacia* species’ mean wood density was 0.39 g/cm^3^, ranged from 0.12 to 0.63 g/cm^3^. In this study, wood density was recognized as a predicting variable to test AGB predictions and tree components. However, it did not improve the performance of models and were not significantly different in the biomass estimation (*p* = *0.0091*) in all tested models. This is supported by findings from other researches such as that of Lisboa et al. [20] and Paul et al. [68].

The leaf size of the *Acacia* species is very small, compared to many other broad-leaved tree species and, hence it is not expected to have substantial contributions to the total biomass estimation. The moisture content of the leaves is high and the dry weight is understandably low. This implies that the contribution of leaves for the total AGB is minimal which is helpful to select the simplest model in our study area. This is supported by Mate [69], the contribution of leaves to the total AGB was only 3% in the Miombo woodlands of Mozambique.

While the biomass estimates based on our equation significantly differed from those based on Brown [58] and Chave et al. [15], possibly, the observed biomass significant differences could arise from the fact that the Omo Gibe *Acacia* woodland ecosystem is different from the Chave et al. and Brown models development ecosystem [31], on the other hand, the model developed using data Henry et al. [31] from the dry forest of East Africa and Guedes et al. [59] equations, which were developed in the dry woodland ecosystem of Mozambique were more similar to our study area (at least from climate perspectives). No statistically significant difference (p > 0.05) and less error were observed among the allometric model developed in the Omo Gibe woodland and those by Henry et al. [31] and Guedes et al. [59]. Moreover, Henry had taken vegetation data from Ethiopia during model development and the Guedes model was also used widely in the dryland forest of Africa. The results of RMPE and MPE analysis indicate that the model developed from Mozambique woodlands and East Africa dry forest can predict the AGB in woodlands of Omo Gibe in Ethiopia. This could be due to having a similar agro-ecological zone of the equation’s developments.

Soromessa [70] also reported the overestimation of biomass when Brown [58] was applied to their observed data of *Juniperus procera* (Hochst. Ex. Endl.) and *Podocarpus falcatus* (Thunb.) Mirb. in Ethiopia. The findings of Wondrade et al. [71], and Tesfaye et al. [18] are also in agreement with our study. Yet, Chave et al. [15] and Brown [58] allometric equations are widely used in Ethiopia for carbon stock assessment and they resulted in uncertainty and unreliable estimation of the biomass [71].

The findings from these comparisons imply that the choice of an appropriate allometric equation in a given set of environmental conditions and tree species is essential for accurate estimating the AGB of a tree and generating reliable information. Studies also showed that provincial differences in tree diameter and height are important sources of uncertainties in estimating biomass using allometric equations [72]. The sample trees used by Brown [58] and Chave et al. [15] were, for instance, typically tall trees and large diameter size that may grow much more than 20 m in the moist evergreen forest, while woodland in Omo Gibe of southwest Ethiopia is dominated by lower height and diameter species, which results in lower AGB for trees in the woodlands. It is also evident that both Brown [58] and Chave et al. [15] sample data were not collected from within Ethiopian woodland areas. Thus, site-specific models need to be developed to obtain a reliable allometric equation for biomass assessment and carbon stock estimates for use in a wide range of environmental and ecological applications, including REDD+. Hence, the accuracy of carbon sequestration potential estimation for Ethiopian forests using site-specific allometric could be improved if allometric equations are rigorously tested and developed for the major forest ecosystems of the country.

The allometric equation developed in this study could be a substantial contribution to efforts being made to improve the accuracy of biomass and carbon sequestration estimates of *Acacia* woodland in Ethiopia. The equation may also be usable in other similar ecosystems elsewhere, particularly in data-scarce regions and where robust allometric equations are lacking. However, the performance of the models in other similar ecosystems of *Acacia* woodland may need to be further tested. Future studies may also consider the influence of environmental variables such as climate and topography on the performances of different models and the influence of different predictor variables on AGB.

## Conclusion

The study has developed site- and species-specific models for estimating AGB of *Acacia* species, particularly the two genera (*Vachellia* and *Senegalia*), in Omo Gibe woodland of Ethiopia. The newly developed models can be used for generating reliable information on carbon stock estimation and Monitoring, Reporting, and Verification (MRV) component of REDD+ particularly, in *Acacia* woodlands of Ethiopia. The model based on DBH alone as predictor variables and a combination of DBH and H was found to be better performing models in predicting AGB, compared to the other alternative models. The power model with DBH alone was shown to perform best in terms of model performance measurement (AIC, RMSE, RMPE, and MPE). Conversely, the model based on height combined with DBH was found to be the second better predictor variable. Wood density did not improve the performance of all tested models. Therefore, the finding did not encourage the use of multiple predictor variables for the estimation of AGB of *Acacia* species. The costs and time involved in measuring several variables are also essential in choosing an appropriate allometric equation. Moreover, the site-specific model is a precise estimator of AGB since it considers the environmental factors and the semi-destructive method can apply in the wider area of a forest, degraded land, threaten species and cutting prohibited areas of the forest. In sum, our study concludes that the inclusion of multiple predicting variables may not necessarily lead to considerable improvements for predicting the AGB but a single variable such as DBH may provide biomass estimates at a sufficient accuracy level.

## Supplementary Information

**Additional file 1: Table S1.** Tree parameter values, total AGB, and components biomass for each sample tree. **Table S2.** Equation coefficients, and model goodness-of-fit performance statistics for estimating AGB of Acacia species biomass. **Table S3.** Correlation between explanatory variables (correlation coefficient).

## Data Availability

All authors declare that the data used in this research are available upon request. The datasets generated and analyzed during the current study are available from the corresponding author on reasonable request.
