# Supplementary material for: Allometric equations for selected Acacia species (Vachellia and Senegalia genera) of Ethiopia
Source: Carbon Balance Manag. 2021 Nov 2;16:34. doi: 10.1186/s13021-021-00196-1 (PMC8561847; doi:10.1186/s13021-021-00196-1)
Supplement: Supplementary file 1 — Additional file 1: Table S1. Tree parameter values, total AGB, and components biomass for each sample tree. Table S2. Equation coefficients, and model goodness-of-fit performance statistics for estimating AGB of Acacia species biomass. Table S3. Correlation between explanatory variables (correlation coefficient). [file 13021_2021_196_MOESM1_ESM.docx]

**Supplementary Materials**

Allometric equations for Ethiopian selected Acacia species (*Vachellia*

and *Senegalia* genera) of Ethiopia Abreham Berta Aneseyee^1^* Teshome Soromessa^2^, Eyasu Elias^2^ and Gudina Legese Feyisa^2^

^1^College of Agriculture and Natural Resource, Department of Natural Resource Management, Wolkite University, Wolkite, P. O. Box 07, Wolkite, Ethiopia.

^2^Center for Environmental Science, College of Computational and Natural Science, Addis Ababa University, P. O. Box No: 1176, Addis Ababa, Ethiopia.

*Corresponding author: [abresh1240@gmail.com](mailto:abresh1240@gmail.com), [abreham.berta@wku.edu.et](mailto:abreham.berta@wku.edu.et)

Table S1. Tree parameter values, total AGB, and components biomass for each sample tree

| Genera | DBH (cm) | H (m) | Volume (m^3^) | Untrimmed SB* branch mean diameter (cm) | Untrimmed SB branch mean length (m) | Trimmed SB mean length (m) | Density (g/cm^3^) | Leaf biomass (kg/tree) | Trimmed biomass (kg/tree) | Untrimmed SB diameter branch (cm) | Untrimmed SB branch biomass (kg/tree) | Dry section (kg/tree) | AGB (kg/tree) |
| --- | --- | --- | --- | --- | --- | --- | --- | --- | --- | --- | --- | --- | --- |
| *Polyacantha* | 95 | 9 | 5 | 15 | 7 | 3 | 0.37 | 0.27 | 1.23 | 47 | 5.85 | 1850 | 1857.35 |
| *Polyacantha* | 84 | 8 | 5.5 | 11 | 8 | 4 | 0.5 | 0.16 | 1.12 | 32 | 4.73 | 2750 | 2756.01 |
| *Polyacantha* | 123 | 22 | 9 | 16 | 7 | 3 | 0.4 | 0.04 | 1 | 32 | 4.26 | 3600 | 3605.3 |
| *Polyacantha* | 90 | 10 | 6 | 14 | 4 | 2 | 0.4 | 0.07 | 1.03 | 27 | 4.41 | 2400 | 2405.51 |
| *Polyacantha* | 85 | 9 | 6 | 15 | 3 | 2 | 0.4 | 0.09 | 1.05 | 33 | 7.57 | 2400 | 2408.71 |
| *Polyacantha* | 155 | 22 | 12 | 18 | 12 | 5 | 0.35 | 0.17 | 1.13 | 55 | 9.73 | 4200 | 4211.03 |
| *Polyacantha* | 100 | 15 | 8 | 16 | 5 | 1 | 0.32 | 0.15 | 1.11 | 47 | 8.84 | 2560 | 2570.1 |
| *Polyacantha* | 75 | 7 | 6 | 11 | 3 | 3 | 0.3 | 0.03 | 0.99 | 10 | 6.74 | 1800 | 1807.76 |
| *Polyacantha* | 115 | 13 | 8 | 19 | 7 | 3 | 0.42 | 0.22 | 1.18 | 66 | 11.23 | 3360 | 3372.63 |
| *Polyacantha* | 95 | 11 | 7 | 12 | 4 | 3 | 0.33 | 0.1 | 1.06 | 23 | 6.16 | 2310 | 2317.32 |
| *Polyacantha* | 70 | 8 | 6.5 | 11 | 6 | 2 | 0.45 | 0 | 0.96 | 11 | 9.56 | 2925 | 2935.52 |
| *Polyacantha* | 77 | 9 | 4.5 | 12 | 4 | 3 | 0.35 | 0.12 | 1.08 | 51 | 7.82 | 1575 | 1584.02 |
| *Polyacantha* | 134 | 21 | 10 | 19 | 7 | 3 | 0.33 | 0.16 | 1.12 | 44 | 7.45 | 3300 | 3308.73 |
| *Polyacantha* | 112 | 16 | 10 | 13 | 13 | 5 | 0.2 | 0.05 | 1.01 | 36 | 5.98 | 2000 | 2007.04 |
| *Polyacantha* | 95 | 10 | 9 | 12 | 5 | 1 | 0.24 | 0.01 | 0.95 | 10 | 4.8 | 2160 | 2165.76 |
| *Polyacantha* | 90 | 8 | 9 | 11 | 6 | 2 | 0.34 | 0.01 | 0.97 | 7 | 6.62 | 3060 | 3067.6 |
| *Polyacantha* | 135 | 21 | 16 | 19 | 16 | 4 | 0.29 | 0.11 | 1.07 | 34 | 7.41 | 4640 | 4648.59 |
| *Polyacantha* | 80 | 11 | 8 | 13 | 9 | 4 | 0.43 | 0.07 | 1.03 | 23 | 9.59 | 3440 | 3450.69 |
| *[Average]* | 101 | 13 | 8 | 14 | 7 | 3 | 0 | 0 | 1 | 33 | 7 | 2796 | 2804 |
| *etbaica* | 58 | 7 | 4 | 11 | 3 | 3 | 0.4 | 0.2 | 1.16 | 12 | 2.68 | 1600 | 1604.04 |
| *etbaica* | 45 | 6 | 4 | 11 | 4 | 2 | 0.5 | 0.21 | 1.17 | 24 | 4.02 | 2000 | 2005.4 |
| *etbaica* | 55 | 7.5 | 4 | 11 | 6 | 2 | 0.6 | 0.1 | 1.06 | 20 | 5.5 | 2400 | 2406.66 |
| *etbaica* | 130 | 19 | 6 | 19 | 6 | 3 | 0.63 | 0.07 | 1.03 | 23 | 7.35 | 3780 | 3788.45 |
| *etbaica* | 94 | 9 | 4 | 13 | 4 | 3 | 0.25 | 0.23 | 1.19 | 10 | 8.67 | 1000 | 1010.09 |
| *etbaica* | 41 | 5.5 | 3.5 | 10.5 | 4 | 3 | 0.53 | 0.23 | 1.19 | 11 | 6.31 | 1855 | 1862.73 |
| *etbaica* | 115 | 10.5 | 6 | 13 | 5 | 1 | 0.29 | 0.1 | 1.06 | 14.5 | 7.23 | 1740 | 1748.39 |
| *etbaica* | 77 | 8.5 | 5 | 12 | 6 | 2 | 0.37 | 0.09 | 1.05 | 17.34 | 5.48 | 1850 | 1856.62 |
| *etbaica* | 72 | 8 | 5 | 13 | 3 | 2 | 0.5 | 0.07 | 1.03 | 23 | 8.21 | 2500 | 2509.31 |
| *etbaica* | 56 | 6.5 | 4 | 14 | 5 | 1 | 0.27 | 0.11 | 1.07 | 22 | 9.19 | 1080 | 1090.37 |
| *etbaica* | 110 | 11 | 7 | 17 | 2 | 2 | 0.45 | 0.09 | 1.05 | 13 | 10.54 | 3150 | 3161.68 |
| *etbaica* | 57 | 13 | 4 | 15 | 5 | 1 | 0.57 | 0.1 | 1.06 | 9 | 10.34 | 2280 | 2291.5 |
| *[Average]* | 76 | 9 | 5 | 13 | 4 | 2 | 0 | 0 | 1 | 17 | 7 | 2103 | 2111 |
| seyal | 48 | 6 | 4 | 11 | 4 | 2 | 0.44 | 0.15 | 1.11 | 7 | 1.2 | 1760 | 1762.46 |
| seyal | 132 | 19 | 6 | 19 | 9 | 5 | 0.58 | 0.32 | 1.28 | 10 | 1.71 | 3480 | 3483.31 |
| seyal | 87 | 10 | 6 | 13 | 7 | 3 | 0.4 | 0.08 | 1.04 | 23 | 0.59 | 2400 | 2401.71 |
| seyal | 76 | 9 | 5 | 12 | 5 | 1 | 0.6 | 0.14 | 1.1 | 34 | 1.2 | 3000 | 3002.44 |
| seyal | 134 | 13 | 7 | 18 | 3 | 4 | 0.18 | 0.09 | 1.05 | 43 | 0.76 | 1260 | 1261.9 |
| seyal | 105 | 12 | 7 | 15 | 4 | 3 | 0.33 | 0.24 | 1.2 | 16 | 2.62 | 2310 | 2314.06 |
| seyal | 85 | 8 | 5 | 12 | 7 | 3 | 0.25 | 0.09 | 1.05 | 14 | 1.1 | 1250 | 1252.24 |
| seyal | 125 | 20 | 8 | 17 | 8 | 4 | 0.33 | 0.18 | 1.14 | 29 | 1.34 | 2640 | 2642.66 |
| seyal | 95 | 8 | 7 | 12 | 4 | 3 | 0.29 | 0.1 | 1.06 | 15 | 0.61 | 2030 | 2031.77 |
| seyal | 39 | 9 | 3.5 | 11 | 6 | 2 | 0.4 | 0.18 | 1.14 | 10 | 1.58 | 1400 | 1402.9 |
| seyal | 40 | 9 | 4 | 12 | 3 | 2 | 0.35 | 0.13 | 1.09 | 38 | 1.07 | 1400 | 1402.29 |
| *[Average]* | 88 | 11 | 6 | 14 | 5 | 3 | 0 | 0 | 1 | 22 | 1 | 2085 | 2087 |
| *tortilis* | 77 | 7 | 5 | 13 | 6 | 3 | 0.21 | 0.14 | 1 | 21 | 1 | 1050 | 1052.372 |
| *tortilis* | 29 | 6 | 5 | 12 | 5 | 1 | 0.3 | 0.16 | 1.12 | 15 | 1.14 | 1350 | 1352.42 |
| *tortilis* | 65 | 8 | 4 | 11 | 3 | 2 | 0.12 | 0.17 | 1.13 | 12 | 1.17 | 480 | 482.47 |
| *tortilis* | 110 | 15 | 9 | 18 | 5 | 1 | 0.39 | 0.61 | 1.57 | 37 | 4.02 | 3510 | 3516.2 |
| *tortilis* | 32 | 6 | 5 | 11 | 5 | 1 | 0.58 | 0.78 | 1.74 | 47 | 5.91 | 2900 | 2908.43 |
| *tortilis* | 96 | 7 | 7 | 12 | 7 | 3 | 0.36 | 0.4 | 1.36 | 25 | 3.68 | 2520 | 2525.44 |
| *tortilis* | 143 | 17 | 10 | 19 | 4 | 2 | 0.5 | 0.78 | 1.74 | 47 | 4.22 | 5000 | 5006.74 |
| *tortilis* | 112 | 13 | 8 | 17 | 6 | 2 | 0.4 | 0.64 | 1.6 | 39 | 6.34 | 3200 | 3208.58 |
| *tortilis* | 47 | 7 | 4 | 11 | 5 | 1 | 0.18 | 0.14 | 1.1 | 10 | 0.79 | 720 | 722.03 |
| *tortilis* | 87 | 12 | 8 | 14 | 5 | 2 | 0.3 | 0.56 | 1.52 | 34 | 1.85 | 2400 | 2403.93 |
| *tortilis* | 73 | 8 | 6 | 13 | 2 | 2 | 0.55 | 0.64 | 1.6 | 39 | 2.11 | 3300 | 3304.35 |
| *tortilis* | 67 | 8 | 5 | 12 | 4 | 2 | 0.5 | 0.66 | 1.62 | 40 | 3.61 | 2500 | 2505.89 |
| *tortilis* | 64 | 11 | 5 | 11 | 4 | 3 | 0.25 | 0.33 | 1.29 | 21 | 2.73 | 1250 | 1254.35 |
| *[Average]* | 77 | 10 | 6 | 13 | 5 | 2 | 0 | 0 | 1 | 30 | 3 | 2322 | 2326 |

NB: Genera of *Vachellia= etbaica, Seyal* and *tortilis ;* Genera of *Senegalia* = *Polyacantha*

*SB*=Short branch; the trimmed short branch and untrimmed short branch can be differentiated based on their diameter. A trimmed small branch, having diameter < 10 cm were harvested for the analysis and untrimmed branch (10 to 20cm) were not cut down, but the biomass was determined based on a regression equation using biomass of trimmed small branch and untrimmed diameter measurement.*

Table S2. Equation coefficients, and model goodness-of-fit performance statistics for estimating AGB of *Acacia* species biomass

|  | **Statistical parameter** | | | | **Model Performance statistic** | | | |
| --- | --- | --- | --- | --- | --- | --- | --- | --- |
| **Alternative model** | **Α** | **β_1_** | **β_2_** | **β_3_** | **AIC** | **RSE** | **MPE** | **RMPE** |
| **Total above-ground biomass** | | |  |  |  |  |  |  |
| model 1 ^a^ | 0.5099*** | 1.9141^***^ | |  | 494 | 52 | 1.74 | 0.06 |
| model 2 ^b^ | 0.2450*** | 1.4210^***^ | -0.7996*** | | 501 | 61 | 1.81 | 0.08 |
| model 3 | 0.3215* | 1.3765^***^ | 0.1137^ns^ | | 589 | 90 | 2.90 | 0.21 |
| model 4 | 0.1345^**^ | 1.4213^***^ | -0.1507** | 0.1432^ns^ | 581 | 83 | 2.51 | 0.17 |
| model 5 | 0.76452* | 1.20956^ns^ | |  | 582 | 84 | 2.55 | 0.19 |
| model 6 | 0.2145^**^ | -1.1856*** | |  | 525 | 74 | 2.01 | 0.12 |
| **Dry section** | |  |  |  |  |  |  |  |
| model 1 | 0. 25412*** | 1.24450^***^ | |  | 329 | 39 | 2.91 | 0.44 |
| model 2 | 0.2981*** | -0.1324^***^ | 0.2365^***^ | | 334 | 45 | 2.99 | 0.9 |
| model 3 | 0.8431* | 0.2754^***^ | 0.8223^ns^ |  | 381 | 69 | 3.06 | 0.8 |
| model 4 | 0.1867^**^ | 0.6143^***^ | 0.3414^***^ | 0.891 ^ns^ | 350 | 54 | 4.01 | 0.75 |
| model 5 | 0.4590* | 0.6011^ns^ | |  | 374 | 60 | 4.05 | 0.71 |
| model 6 | 0.3512^**^ | -0.1312^***^ | |  | 337 | 51 | 3.58 | 0.68 |
| **Untrimmed small branch** | | |  |  |  |  |  |  |
| model 1 | 0.26366^***^ | 1.06391^***^ | |  | 199 | 3 | 4.25 | 0.94 |
| model 2 | 0.26366*** | 1.06391^***^ | 0.2586^***^ | | 201 | 3 | 4.50 | 1.04 |
| model 3 | 0.54527* | -1.02274^***^ | 0.06779^ns^ | | 229 | 5 | 6.00 | 1.41 |
| model 4 | 0.65273** | 1.41987^***^ | 0.44167*** | 0.57569^ns^ | 213 | 4 | 5.25 | 1.40 |
| model 5 | 0.65273* | 1.45895 ^ns^ | |  | 221 | 5 | 5.50 | 1.35 |
| model 6 | 0.54527** | -1.02274^***^ | |  | 205 | 4 | 5.00 | 1.31 |
| **Trimmed small branch** | | | |  |  |  |  |  |
| model 1 | 0.095766^***^ | 0.006662^***^ | |  | 28 | 0.1 | 4.75 | 1.03 |
| model 2 | -0.1211** | 0.019117 ^ns^ | 0.08232^ns^ | | 32 | 0.2 | 4.75 | 1.13 |
| model 3 | 0.285117^ns^ | -0.00868*** | -0.4148^ns^ | | 49 | 1.2 | 6.25 | 1.51 |
| model 4 | 0.2155547^ns^ | -0.00519^***^ | 0.0454902 ^ns^ | -0.869ns | 42 | 0.3 | 5.50 | 1.49 |
| model 5 | 0.2155547 | -0.00815^ns^ | |  | 46 | 0.3 | 6.00 | 1.44 |
| model 6 | -0.12118^**^ | 0.082320^***^ | |  | 37 | 0.2 | 5.50 | 1.40 |
| **Trimmed leaves** | | | |  |  |  |  |  |
| model 1 | 0.006754 ^ns^ | 0.054671 ^ns^ | |  | 9 | 0.0 | 6.67 | 1.55 |
| model 2 | -0.1432^**^ | 0.027416 ^ns^ | 0.039847^**^ | | 12 | 0 | 8.33 | 1.65 |
| model 3 | 0.03454^ns^ | -0.09419 ^ns^ | -0.3461^ns^ | | 30 | 0.2 | 10.33 | 1.98 |
| model 4 | 0.12367^ns^ | -0.07540^ns^ | 0.02975^*^ | -0.6575 ^ns^ | 23 | 0.1 | 9.33 | 1.97 |
| model 5 | 0.03437** | -0.0456^*^ |  |  | 26 | 0.1 | 9.67 | 1.96 |
| model 6 | -0.09823^**^ | 0.03412* | |  | 19 | 0.1 | 9.00 | 1.92 |

**p≤0.05; **p≤0.01; ***p≤0.001, ns= statistically non-significant, a and b are selected Equation for further biomass analysis. Dry section = stem and large branch biomass (kg); Untrimmed small branch = biomass of small branches (diameter between 10 and 20 cm) (kg); Trimmed small branch = biomass of small branches (diameter <10 cm) (kg); Trimmed leaves = the biomass of trimmed leaves (kg); Total above-ground biomass = dry section + untrimmed small branches + trimmed branch + leaves biomass (kg); β_1_, β_2,_ and β_3_ are coefficients for each predictor variable, α is parameters intercept of the models, RSE = mean residual standard error.*

Table S3: Correlation between explanatory variables (correlation coefficient)

|  | **DBH** | **H** | **Wood density** |
| --- | --- | --- | --- |
| **DBH** | 1.000 | 0.835 | 0.026 |
| **H** | 0.835 | 1.000 | 0.001 |
| **Wood density** | 0.026 | 0.001 | 1.000 |
